# Development of a Photocrosslinkable Collagen–Bone Matrix Hydrogel for Bone Tissue Engineering

**DOI:** 10.3390/polym17070935

**Published:** 2025-03-29

**Authors:** Po-Hsun Chen, Wei-Bor Tsai

**Affiliations:** Department of Chemical Engineering, National Taiwan University, No. 1, Sec. 4, Roosevelt Rd., Taipei 106, Taiwan; f10524114@ntu.edu.tw

**Keywords:** modified collagen, bone matrix, hydrogel, hydroxyapatite, osteogenesis, mineralization

## Abstract

Bone tissue engineering aims to restore lost bone and create an environment conducive to new bone formation. To address this challenge, we developed a novel biomimetic hydrogel that combines maleic anhydride–modified type I collagen (ColME) with maleic anhydride–modified demineralized and decellularized porcine bone matrix particles (mDBMp), forming a composite ColME–mDBMp (CMB) hydrogel. Chemical modification of collagen resulted in a high degree of substitution, thereby enhancing its photocrosslinkability. Integration of mDBMp into the ColME hydrogel via photocrosslinking resulted in enhanced physiological stability, reduced shrinkage, and improved mechanical strength compared to gelatin methacrylate (GelMA)-based hydrogels. Moreover, mineralization of the CMB hydrogel promoted the formation of pure hydroxyapatite (HAp) crystals, providing superior stiffness while maintaining ductility relative to GelMA-based hydrogels. In vitro, human bone marrow mesenchymal stem cells (hBMSCs) encapsulated in CMB hydrogels exhibited enhanced proliferation, cell–matrix interactions, and osteogenic differentiation, as evidenced by increased calcium deposition and histological analysis. These results demonstrate that the CMB hydrogel, enriched with extracellular matrix (ECM) components, shows considerable promise over current GelMA-based hydrogels for bone tissue engineering.

## 1. Introduction

Bone defects resulting from surgery, injury, or disease often result in critical-sized bone defects that do not heal fully without surgical intervention [1]. Though autologous bone grafts are regarded as the gold standard, limitations including donor site morbidity and availability underline the urgency of alternative strategies [2,3]. Clinically, synthetic substitutes (metallic [4,5], carbon-based [6,7], ceramic [8,9], and polymer-based filler [10,11]) offer advantages, such as low immunogenicity and reproducible quality, but often lack integration with host bone, do not closely mimic the hierarchical structure of bone, or degrade sub-optimally. Consequently, there is growing interest in natural extracellular matrix (ECM)-based alternatives that not only provide structural support but also dynamically influence host bone cell behavior, facilitating bone homeostasis and efficient repair.

Bone’s organic ECM is predominantly composed of type I collagen, which accounts for approximately 90% of its content and serves as an important structural protein. These collagen fibrils form a hierarchical architecture by integrating non-collagenous proteins into fibers, thereby contributing substantially to mechanical strength [12]. Because collagen contains integrin-binding motifs supporting cell adhesion, proliferation, and initiation of mineralization, it has attracted considerable attention as a natural bone substitute [13]. Hydrogels, which possess porosity comparable to native ECM, have been extensively investigated for bone tissue engineering [14]. However, collagen-only hydrogels often exhibit low mechanical strength when fully hydrated, necessitating additional polymers or advanced crosslinking strategies [15]. To date, numerous chemically modified collagen formulations have been developed to improve neutral solubility and photocrosslinkability, thereby providing sufficient mechanical properties and introducing innovative treatments for bone substitution [16,17,18,19,20]. By contrast, gelatin methacrylate (GelMA), the modified product of denatured collagen, has been widely applied for bone repair due to its biocompatibility, modifiability, and crosslinkability [19,21]. Nevertheless, hydrogels derived from modified collagen have been shown to promote enhanced osteogenesis compared to GelMA, even in the absence of additional differentiation inducers [19]. Additionally, several studies have reported that collagen-based hydrogels generally exhibit higher bioactivity than gelatin-based hydrogels, owing to the numerous ligand sites present within collagen’s helical structure [22,23,24]. Thus, advanced ECM hydrogels from collagen serve as an attractive option for bone tissue regeneration because of their tunable properties and bioactive nature.

Although collagen can modulate cellular behavior, it is not inherently osteo-inductive. To enhance osteo-inductivity, demineralized bone matrix, which is rich in bone morphogenetic proteins and growth factors, has been integrated into hydrogel systems [25,26]. Complete or near-complete demineralization is essential to remove minerals and genetic materials while exposing endogenous growth factors, enhancing efficacy and reducing immunogenicity [27]. As reported, GelMA-only hydrogels were limited in bone regeneration when implanted in calvarial defects [28]. In contrast, composite hydrogels incorporating demineralized bone matrix particles into GelMA have demonstrated improved osteogenic differentiation and new bone formation [29]. Moreover, collagen-only hydrogels have been shown to exhibit insufficient osteogenic differentiation compared to hydrogels derived from demineralized bone matrix [30,31]. Hence, encapsulating allografts or xenografts in an osteoconductive ECM hydrogel is considered a promising strategy.

Osteoblast-mediated osteogenesis proceeds through cell–cell and cell–ECM interactions that initiate mineralization, culminating in mature bone. Because bone naturally mineralizes via soft collagen fibrils and stiff hydroxyapatite (HAp) minerals, replicating this process in functionalized hydrogels is a pivotal strategy to expedite bone repair. Hydrogel immersion-based mineralization effectively deposits HAp with varying crystallinity, thereby enhancing stiffness and cell proliferation [32,33]. More recently, enzymatic mineralization via alkaline phosphatase (ALP) has demonstrated in situ integration of HAp into hydrogels, using anionic polymer nucleation sites for ion diffusion control [34]. This design enables optimized control of ion diffusion, resulting in a relatively uniform and complete deposition. Such integration of organic–inorganic components within hydrogel offers new avenues in ECM hydrogel design.

Our previous work demonstrated that maleic anhydride-modified type I collagen (ColME) can achieve a DS above 90% while retaining its secondary structure and favorable degradation properties, with robust biocompatibility in vitro and in vivo [35,36]. Building on this, the present study combines ColME with maleic anhydride-modified demineralized and decellularized porcine bone matrix particles (mDBMp) to form a composite ColME–mDBMp (CMB) hydrogel for bone tissue engineering. By innovatively modifying the bone matrix particles, additional alkene groups are introduced, enabling enhanced photocrosslinking with the hydrogel precursor. Because demineralized bone matrix has long been utilized as a filler for bone defects, integrating it in a photocrosslinkable hydrogel network provides both osteogenesis and mechanical advantages. Specifically, the additional carboxylic groups in ColME promoted calcium uptake and mineralization, while mDBMp increased crosslinking between the bone matrix and the hydrogel in CMB, yielding prolonged physiological stability and enhanced mechanical performance relative to GelMA–mDBMp (GMB) hydrogel. Furthermore, upon mineralizing both CMB and GMB hydrogels, CMB more effectively facilitate HAp synthesis. Finally, a series of in vitro analyses, including encapsulation of human bone marrow mesenchymal stem cells (hBMSCs) and histochemical staining, confirmed the hydrogel’s capacity to support cell adhesion, proliferation, and osteogenesis.

## 2. Materials and Methods

### 2.1. Materials

Bovine type I collagen (atelocollagen) was sourced from Guangdong Victory Biotech (Foshan, China). Femur and tibia of porcine were purchased from a local market. Gelatin type A from porcine skin, acetone, ethanol, dimethyl sulfoxide (DMSO), hydrogen peroxide, magnesium chloride hexahydrate, ethylenediaminetetraacetic acid (EDTA), trizma hydrochloride (Tris-HCl), deoxyribonuclease I from bovine (DNase), ribonuclease A from bovine (RNase), sodium azide, trinitro-benzene-sulfonic acid (TNBS), sodium bicarbonate, glycine, lithium phenyl-2,4,6-trimethylbenzoylphosphinate (LAP), dexamethasone, L-ascorbic acid 2-phosphate, B-glycerophosphate disodium salt hydrate, papain, L-cysteine dihydrochloride, calcium chloride, sodium phosphate dibasic, Alizarin Red S, paraformaldehyde, hematoxylin solution, eosin Y, DPX mountant, hydroxyapatite nano-powder (<200 nm), and sucrose were purchased from Sigma–Aldrich (Burlington, VT, USA). Acetic acid was purchased from Honeywell Fluka (Charlotte, NC, USA). Maleic anhydride was purchased from Alfa Aesar (Ward Hill, MA, USA). N-succinimidyl methacrylate was purchased from Tokyo Chemical Industry (Tokyo, Japan). Tween-20 was purchased from Zymeset (Taipei, Taiwan). Calcium Assay (Arsenazo III) was purchased from Sekisui (Burlington, VT, USA). Calcium chloride dihydrate, Tris (Base), and xylene were purchased from J.T. Baker (Phillipsburg, NJ, USA). Minimum Essential Medium Alpha Medium (α-MEM, no nucleosides, powder) was purchased from Gibco (Grand Island, NE, USA). Fetal bovine serum (FBS) was purchased from Peak Serum (Wellington, FL, USA). Antibiotic/Antimycotic solution (100×) was purchased from Capricorn (Ebsdorfergrund, Germany). LIVE/DEAD cell imaging kit and PicoGreen dsDNA assay kit were purchased from Invitrogen (Waltham, MA, USA). Optimal cutting temperature (OCT) compound was purchased from Fisher HealthCare (Waltham, MA, USA).

### 2.2. Synthesis and Characterization of ColME and GelMA

Maleic anhydride–modified collagen (ColME) was synthesized from bovine type I collagen following a previously described method [35]. Briefly, collagen was dissolved in 0.5 M acetic acid to achieve a 0.15% *w*/*v* (hereafter abbreviated as 0.15%) solution, and the pH was gradually raised to 8.5 using NaOH solution (10 M). A solution of maleic anhydride in acetone was then added at an equivalent amount to collagen. This mixture was incubated at 4 °C for 24 h with constant gentle stirring. The resulting ColME was precipitated using HCl solution (6 M), then dialyzed against phosphate-buffered saline (PBS) at 4 °C for 3 days, followed by an additional 1 day in deionized water. The final product was freeze-dried and stored at −20 °C. Collagen concentrations were measured by a hydroxyproline assay (details in S1 of the Supplementary Materials). The degree of substitution (DS) of ColME was determined by quantifying the free amino groups using the TNBS colorimetric assay. Briefly, 100 µL of 0.1% collagen or ColME solution was adjusted to pH 9.0, diluted with 800 µL of 0.1 M sodium bicarbonate buffer (pH 9.8), and then mixed with 800 µL of 0.01% TNBS. After incubation at 37 °C for 1 h in the dark, the absorbance was recorded at 353 nm. The concentration of free amino groups in both collagen and ColME samples was calculated using a standard curve generated from known concentrations of glycine (0 to 2.5 mM). The DS was calculated using the following equation

$$\mathrm{DS}=(1-\frac{\mathrm{amount}\;\mathrm{of}\;\mathrm{amines}\;\mathrm{on}\;\mathrm{ColME}}{\mathrm{amount}\;\mathrm{of}\;\mathrm{amines}\;\mathrm{on}\;\mathrm{collagen}})\;\times \;100\%$$

Gelatin Methacrylate (GelMA) was synthesized based on a previously reported method [37]. In summary, gelatin type A was dissolved in PBS, while N-succinimidyl methacrylate was prepared in DMSO. A 2% gelatin solution was added dropwise to a 0.5% N-succinimidyl methacrylate solution. The mixture was kept at room temperature for 24 h under gentle stirring. Afterward, the product underwent dialysis against PBS for 3 days and deionized water for additional 1 day before being freeze-dried and stored at −20 °C.

### 2.3. Preparation, Modification, and Characterization of DBMp

A schematic illustration of the DBMp preparation is provided in S2 of the Supplementary Materials. Muscles and tendons were removed from porcine bone using a scalpel. The bones were initially treated with 6% hydrogen peroxide for 2 days to remove connective tissues, and debris. The bones were then crushed, rinsed in deionized water, and demineralized in HCl solution (0.5 M) with daily solution changes for 3 days until the solution became transparent. After immersion in 70% ethanol overnight, the demineralized product was freeze-dried for 3 days, ground, and sieved to obtain particles in the 425–500 µm range.

Decellularization followed an adapted enzymatic protocol [38]. Osmotic shock was performed with hypertonic and hypotonic solutions for 12 h each, with an 8-h wash in PBS containing Tween-20 after each treatment. DNase/RNase digestion was conducted for 24 h, followed by a 12-h wash in the same buffer. The product was then immersed in 0.02% sodium azide for 6 h, washed in deionized water for 12 h, centrifuged, and freeze-dried for 3 days, yielding demineralized and decellularized bone matrix particles (DBMp).

For size distribution analysis, particles were assessed via optical microscopy (Nikon Eclipse TS100, Nikon, Tokyo, Japan). The images were analyzed using NIH ImageJ 1.53t software to calculate the average diameter, assuming a circular shape. Calcium content was quantified using an Arsenazo III calcium assay according to the manufacturer’s protocol. Briefly, dry samples of native bone and demineralized bone matrix particles were weighed. The calcium content was extracted by incubating in 0.6 M HCl with gentle shaking for 24 h. A standard curve was prepared using calcium chloride solutions ranging from 0 to 12 mg/mL. For the assay, 10 µL of each standard or sample was mixed with 990 µL of Arsenazo III reagent for 5 min, and the absorbance was measured at 650 nm. DNA content was measured using the PicoGreen dsDNA assay following the manufacturer’s protocol. Briefly, samples were incubated in a solution containing 0.25 mg/mL papain and 3.2 mg/mL L-cysteine dihydrochloride at 60 °C for 24 h to ensure complete digestion. A standard curve was prepared with dsDNA standards ranging from 0 to 1000 ng/mL. For the assay, 100 μL of each sample was mixed with 100 μL of PicoGreen dye in the dark, and fluorescence was measured at an excitation wavelength of 480 nm and an emission wavelength of 520 nm.

Maleic anhydride–modified DBMp (mDBMp) were synthesized similarly to ColME. Briefly, 300 mg of DBMp was suspended in 100 mL of PBS. The reaction was performed using an equivalent amount of maleic anhydride solution (2 M) to the DBMp. The pH was adjusted to 8.5 using NaOH solution (2 M), and the mixture was incubated at 4 °C for 24 h under gentle stirring. The product was then washed five times with deionized water, freeze-dried, and stored at −20 °C.

### 2.4. Fabrication and Characterization of ColME- and GelMA-Based Hydrogels

For hydrogel preparation, 5% GelMA or 1% ColME was formulated with 0.06% LAP and photocrosslinked using UV irradiation (10 mW/cm^2^) for 1.5 min, forming the respective GelMA or ColME hydrogel. For particle-incorporated hydrogels, DBMp or mDBMp (5%) was added at room temperature prior to photocrosslinking. The formulations were designated as follows: GB (5% GelMA with 5% DBMp), GMB (5% GelMA with 5% mDBMp), CB (1% ColME with 5% DBMp), and CMB (1% ColME with 5% mDBMp) hydrogels.

Hydrogel properties under hydration were assessed by incubating samples in PBS at 37 °C. Water uptake was assessed by freeze-drying the hydrogels, immersing them in PBS, and recording weight changes. Then, the water uptake was expressed as the ratio of wet to dry weight. Volume changes were evaluated by measuring the average height and diameter of the hydrogels over time using NIH ImageJ software, with the volume at each time point normalized to the initial volume.

Compression tests were performed using a tensile testing machine (Nidec–Shimpo, Kyoto, Japan) at a constant rate of 6 mm/min until fracture occurred. The elastic modulus was derived from the linear regression (up to 30% strain) of the stress–strain curve, and maximum stress was noted prior to fracture.

### 2.5. Fabrication and Characterization of Mineralized Hydrogels

To test the mineralization of the hydrogels, GMB and CMB hydrogels underwent alternate immersion in 0.40 M CaCl_2_ and 0.24 M Na_2_HPO_4_ (both in tris base buffer at pH 8.0) for 10 or 20 cycles (30 min each). After each immersion, the hydrogels were washed with deionized water. After every 5 cycles, the hydrogels were flipped to ensure uniform mineralization. Finally, samples were immersed in deionized water for 12 h to remove soluble crystals. Compression tests were conducted as described in Section 2.4.

X-ray diffraction (XRD) analysis was performed on freeze-dried, ground hydrogel samples using a MiniFlex diffractometer (Rigaku, Tokyo, Japan), scanning from 2° to 90° (step size: 0.01°; speed: 10°/min). The resulting patterns were matched with standard hydroxyapatite chemical and brushite reference (ICDD JCPDS#72-0713).

### 2.6. Evaluation of Cell Viability and Proliferation of hBMSCs Encapsulated in Hydrogels

Immortalized human bone marrow mesenchymal stem cells (hBMSCs, passages 15–25, T0523 from Applied biological materials, Richmond, BC, Canada) were used. We examined the cell viability and proliferation of hBMSCs encapsulated in 1% ColME hydrogel precursor and 0.06% LAP at a cell density of 10^6^ cells/mL. Subsequent proliferation assays were conducted using GelMA, ColME, GMB, and CMB hydrogels, each encapsulating hBMSCs at a cell density of 10^6^ cells/mL containing 0.06% LAP. Each hydrogel precursor was photocrosslinked via UV irradiation (10 mW/cm^2^) for 1.5 min to ensure gelation. The cell-laden hydrogels were cultured in α-MEM supplemented with 10% FBS and 1% antibiotic-antimycotic solution for 14 days.

Cell viability was assessed via LIVE/DEAD staining using an inverted fluorescence microscope (IX71, Olympus, Tokyo, Japan). DNA content, serving as an index of cell proliferation, was quantified after PBS washing twice and papain digestion using the PicoGreen dsDNA assay as described in Section 2.3.

### 2.7. Evaluation of Osteogenic Differentiation of hBMSCs Encapsulated in Hydrogels

hBMSCs were encapsulated in each hydrogel formulation at a density of 2 × 10^6^ cells/mL and photocrosslinked using UV irradiation (10 mW/cm^2^) for 1.5 min. The hydrogels were initially cultured in complete medium (α-MEM supplemented with 10% FBS) for 3 days, then switched to osteogenic medium (OM) containing 30 nM dexamethasone, 10 mM β-glycerophosphate, 50 µM L-ascorbic acid 2-phosphate, and 10% FBS. Half of the culture medium was changed every 3 days for 28 days.

Hydrogel appearance was monitored via optical microscopy (Nikon Eclipse TS2, Tokyo, Japan). On day 14, 21, and 28, hydrogels were washed twice with PBS (15 min per wash) and subsequently extracted calcium in 0.6 M HCl under gentle shaking for 24 h. Calcium content was measured using the Arsenazo III calcium assay as described in Section 2.3. Concurrently, hydrogels were fixed in 4% paraformaldehyde at 4 °C, embedded in OCT compound, and cryo-sectioned at a thickness of 20 µm. Histological analysis was conducted using Alizarin Red S (ARS) and hematoxylin and eosin ( H&E) staining.

### 2.8. Statistical Analysis

All statistical analyses were carried out using Prism 9 (GraphPad, Boston, USA). A one-way or two-way ANOVA with Tukey’s multiple comparison test was employed. The symbols *, **, and *** denote *p* < 0.05, 0.01, and 0.001, respectively. Data are presented as mean ± standard deviation.

## 3. Results and Discussion

### 3.1. Physicochemical Characterization of ColME

The degree of substitution (DS) of ColME was determined to be 92 ± 9% across four individual modification batches, indicating extensive substitution of amino groups with anionic carboxylic groups. Our earlier study demonstrated that ColME exhibited isoelectric point (pI) of approximately 3.5 and a more negative zeta potential at higher pH values, thereby improving its solubility at neutral pH compared to native collagen, which has a pI of around 7.7 [35].

### 3.2. Characterization of DBMp

**Figure 1:**
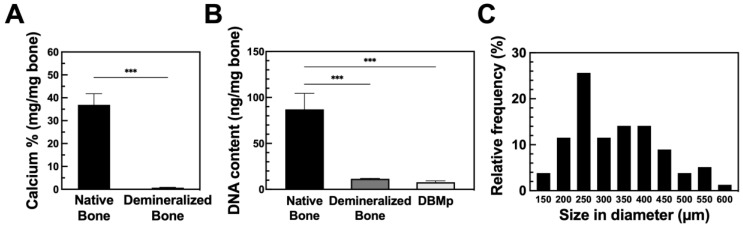
The quantitative analysis of (**A**) residual calcium after demineralization (N = 5), (**B**) DNA content after demineralization and decellularization (N = 4), and (**C**) size distribution (N = 80). All data are shown as mean ± standard deviation; ***, *p* < 0.001.

DBMp was prepared as illustrated in S2 in Supplementary Materials. During demineralization, acid extraction reduced the residual calcium content (based on dry weight) from 37 ± 5% (*w*/*w*) to 0.7 ± 0.2% (*w*/*w*) (Figure 1A). According to guidelines established by the American Association of Tissue Banks (AATB), the residual calcium in demineralized bone should not exceed 8% of the dry weight [39]. Moreover, when the residual calcium content is reduced to less than 1%, the bone becomes radiographically invisible, indicating an adequate degree of demineralization [40]. This level of demineralization is crucial for minimizing potential immune responses, particularly in xenograft applications.

DNA analysis of native bone, demineralized bone, and DBMp revealed 87, 12, and 8 ng per mg of dry weight, respectively (Figure 1B). Most of the DNA was removed during the demineralization process, achieving an 87% reduction. Subsequent decellularization further reduced the DNA content by 4% in the final DBMp product, meeting the criterion of less than 50 ng DNA per mg dry weight for medical applications [41]. Although the removal of cellular debris is essential to minimize immunogenicity, there is a trade-off between sufficient debris removal and preserving the integrity of the extracellular matrix (ECM) [38].

After grinding and sieving through meshes ranging from 425 to 500 µm, the particles exhibited an average size of 331 ± 108 µm (Figure 1C). Notably, the majority of particles were within the 225 to 275 µm range, comprising 26% of the total count. The observed size deviation was primarily attributed to the irregular shapes of the particles. Importantly, the overall particle size did not significantly influence osteo-inductivity [42]. Consequently, DBMp within the size range of 150 to 600 µm were chosen for subsequent experiments.

### 3.3. Fabrication and Characterization of Hydrogel Incorporating DBMp/mDBMp

Incorporating DBMp into hydrogels can potentially improve their mechanical properties. However, conventional DBMp-incorporated hydrogels generally rely on weak physical or ionic interactions between the particles and the hydrogel network. This limited interaction can reduce the mechanical stability and overall integration of DBMp. To address this limitation, we modified DBMp with maleic anhydride to produce mDBMp, which can form rapid covalent crosslinks with the hydrogel precursor through photocrosslinking. This approach reinforces the hydrogel’s mechanical performance. A schematic illustration is provided in S3 in the Supplementary Materials.

**Figure 2:**
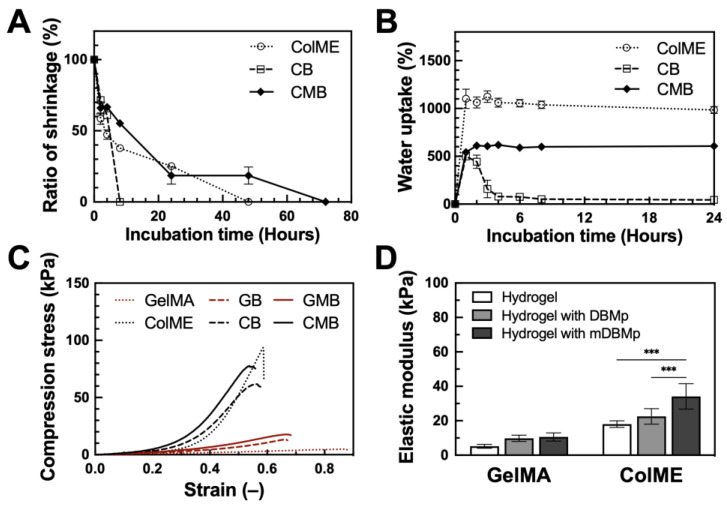
Physiological and mechanical characterization of DBMp/mDBMp-incorporated hydrogel: (**A**) The shrinkage behavior and (**B**) the water uptake capacity of ColME-based hydrogels (N = 3); (**C**) Axial compression test of stress–strain curves and (**D**) quantitative elastic modulus values for GelMA-based versus ColME-based hydrogels with DBMp/mDBMp incorporation (N = 6). All data are shown as mean ± standard deviation; ***, *p* < 0.001.

We first monitored hydrogel shrinkage in ColME, CB, and CMB hydrogels. All ColME-based hydrogels exhibited shrinkage (see S3 in the Supplementary Materials). After 2 h of incubation at 37 °C in PBS, the ColME hydrogel shrank by 42%, whereas CB and CMB hydrogels shrank by 28% and 34%, respectively (Figure 2A). By 8 h, the CB hydrogel dissociated, whereas ColME and CMB hydrogels reached 62% and 45% shrinkage, respectively. The ColME hydrogel continued to shrink until it dissociated after 48 h, while the CMB hydrogel maintained roughly 19% of its initial volume at 24 and 48 h, dissociating at 72 h. This shrinkage phenomenon in collagen-based hydrogels typically arises from elevated temperature and collagen fibril self-assembly [43]. Pre-crosslinking modifications have been shown to mitigate such shrinkage [44]. We also suggest that some non-crosslinked ColME may be lost during incubation, given the varying shrinkage rates among ColME, CB, and CMB hydrogels. Specifically, the CMB hydrogels appear to achieve a higher degree of crosslinking than ColME and CB, thus offering greater resistance to shrinkage. Moreover, unmodified DBMp possibly interfered with the free radical polymerization of the alkene groups on ColME, underscoring the pivotal role of DBMp modification in maintaining hydrogel integrity.

Next, freeze-dried hydrogels were evaluated for water uptake (Figure 2B). After 1 h, the ColME hydrogel displayed the highest water uptake at 1102%, followed by the CMB hydrogel at 542% and the CB hydrogel at 499%. After 24 h, water uptake reached saturation at approximately 1100% for the ColME hydrogel and 600% for the CMB hydrogel. In comparison, the ColME hydrogel fully dissociated by day 2, while the CMB hydrogel remained intact for up to 6 days. These findings demonstrate that freeze-drying enhances the physiological stability of the hydrogels, particularly those incorporating mDBMp.

Finally, we assessed the mechanical properties of the hydrogels. In addition to ColME-based (ColME, CB, CMB) hydrogels, GelMA-based hydrogels were prepared and served as controls: GelMA, GB, and GMB hydrogels. Compression stress–strain curves (Figure 2C) show that 1% ColME-based hydrogels exhibit higher stiffness than 5% GelMA-based hydrogels, as indicated by higher stress and lower strain. Introducing DBMp or mDBMp strengthened both GelMA and ColME systems. Specifically, the elastic modulus of GelMA-based hydrogels increased from the incorporation of particles, reaching 10 ± 2 kPa for the GB hydrogel and 11 ± 2 kPa for the GMB hydrogel (Figure 2D). In ColME-based hydrogels, the corresponding elastic modulus were 23 ± 4 kPa for the CB hydrogel and 34 ± 7 kPa for the CMB hydrogel. These findings indicate that the particles enhance resistance to deformation, with a more pronounced effect in the ColME-based system due to effective crosslinking between the particles and the hydrogel precursor. Furthermore, our ColME hydrogel exhibited superior elastic modulus compared to other photocrosslinkable collagen hydrogels at similar concentrations [19]. Although previous literature reports a reduction in mechanical properties when unmodified particles are incorporated in collagen hydrogels [45], modifying the functional groups on the particles can significantly enhance both the elastic and storage modulus [46]. Therefore, the incorporation of mDBMp is critical for reinforcing the hydrogel network and reducing shrinkage in physiological environments. Notably, ColME-based hydrogels demonstrate superior mechanical properties compared to GelMA-based hydrogels when incorporating mDBMp.

### 3.4. Mineralization of Hydrogels

**Figure 3:**
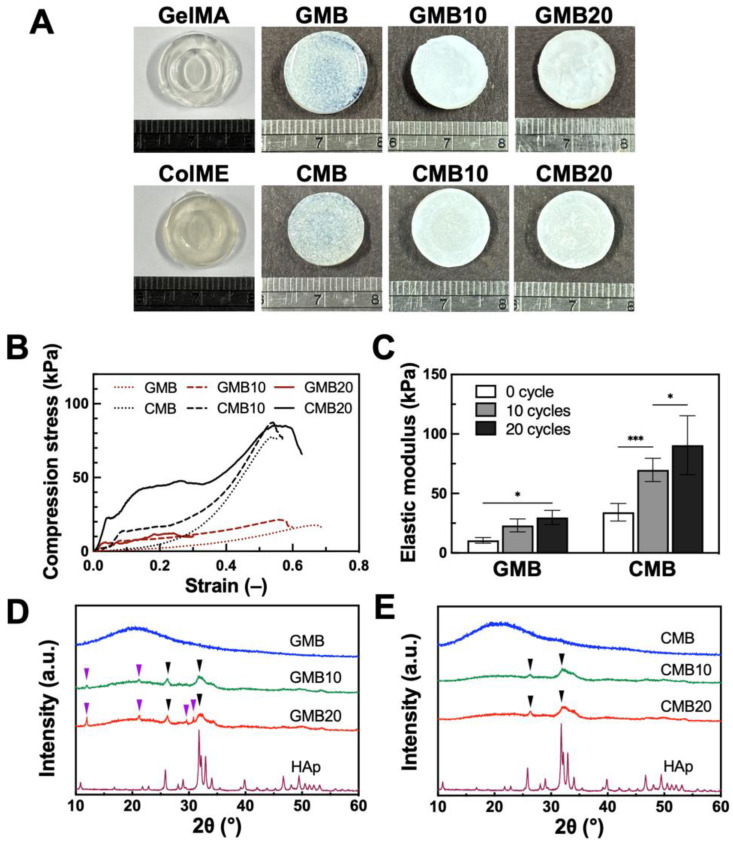
Mineralization of GMB and CMB hydrogels: (**A**) Visual appearance after mineralization cycles; (**B**) Axial compression test of stress–strain curves and (**C**) quantitative elastic modulus value after 10 and 20 mineralization cycles (N = 6); XRD analysis of mineralized (**D**) GMB and (**E**) CMB hydrogels. GMB10 and CMB10 indicate hydrogels with 10 cycles of mineralization. GMB20 and CMB20 indicate hydrogels with 20 cycles of mineralization. Purple arrow: Brushite. Black arrow: HAp; All data are shown as mean ± standard deviation; *, *p* < 0.05, ***, *p* < 0.001.

Phosphate-based bio-ceramics have been extensively applied in bone regeneration strategies, with HAp serving as the principal inorganic component in human bone to support hard tissue. HAp containing hydrogels has demonstrated significantly enhanced mechanical properties and bone repair efficacy, both in conjunction with MSCs and as acellular implants for calvarial bone repair [47]. To emulate this, the hydrogels became more opaque and with white coloration after mineralization, indicative of mineral deposits forming on and within the hydrogel matrix (Figure 3A).

Mechanical properties were then assessed via axial compression testing (Figure 3B). The mineralized GMB hydrogels (GMB10 and GMB20) showed higher stress values but became brittle, exhibiting a pronounced reduction in strain. Conversely, the mineralized CMB hydrogels (CMB10 and CMB20) demonstrated an increase in stress while retaining ductility. This suggests an improved load-bearing and energy–absorbing capacity. Thus, while mineralization strengthened the GMB hydrogel at the expense of ductility, it endowed the CMB hydrogel with both enhanced stiffness and sustained deformability. Quantitative analysis of the elastic modulus is presented in Figure 3C. For GMB hydrogels, the elastic modulus rose by 2.2-fold (to 23 ± 5 kPa) and 2.8-fold (to 30 ± 6 kPa) after 10 and 20 mineralization cycles, respectively. Similarly, the CMB hydrogel exhibited a 2.0-fold increase (to 70 ± 10 kPa) after 10 cycles and a 2.7-fold increase (to 91 ± 25 kPa) after 20 cycles of mineralization. Consequently, CMB hydrogels were approximately three times stiffer than GMB hydrogels at both cycles, indicating a greater overall stiffness in CMB. This approach, which promotes the formation of a denser HAp layer on the hydrogel surface, has been reported to yield fully developed, bone-like tissue within four weeks of implantation, providing significant mechanical support [48]. However, some HAp hydrogel composites lack degradability, hampering osteoblast migration and infiltration. By contrast, ColME-based hydrogels offer both mechanical durability and biodegradability, permitting better HAp integration than GelMA-based hydrogels.

To characterize the crystal structure of mineralized GMB and CMB hydrogels, XRD analysis was employed to elucidate the crystal phases. Unmineralized GMB and CMB hydrogels showed no peaks, indicating no intrinsic crystallinity (Figure 3D,E). After 10 and 20 mineralization cycles, the GMB hydrogels displayed mixed HAp and brushite peaks. The main diffraction peaks of HAp appeared at approximately 26° and 32°, corresponding to the (0 0 2) and (2 1 1) planes, respectively (Figure 3D). Brushite was identified by peaks near 12°, 21°, 29°, and 31°, correlating to the (0 2 0), (1 2 $\overset{-}{1}$), (1 4 $\overset{-}{1}$), and (1 2 1) planes. In contrast, mineralized CMB hydrogels exhibited only the HAp peaks (Figure 3E). Although HAp formation is generally favored in alkaline environments over brushite, a portion of brushite (with a Ca/P ratio of 1) was simultaneously synthesized in our system [49,50]. This variation in crystal type is likely to have resulted from the diffusion and dissolution of brushite within the hydrogels, especially in the gelatin-based matrix [51]. The exclusive HAp formation in CMB hydrogels may be attributed to the higher anionic charge of ColME discussed in Section 3.1, which offers a more attractive network for calcium ions, encouraging a Ca/P ratio close to 1.67 rather than 1 (brushite) [34,35,52]. Collectively, these findings indicate that pure HAp crystals formed more readily in CMB than in GMB hydrogels. The presence of this inorganic component not only enhances stiffness but, in the form of HAp, also has the potential to improve osteogenic penetration and integration at bone defect sites. Upon transplantation, partial HAp dissolution can stimulate stem cell recruitment and promote new bone formation, creating continuity with the host bone [53].

### 3.5. In Vitro Cell Viability and Proliferation in Hydrogels

We initially evaluated the viability and proliferation of hBMSCs encapsulated in the basal ColME hydrogel (see S4 in the Supplementary Materials). LIVE/DEAD staining revealed that most cells remained viable post-photocrosslinking, with increasing live cell density over 14 days. In addition, DNA content analysis showed a 4.1-fold increase from day 0 to day 14, suggesting that the hydrogel curing procedure had minimal adverse effects on hBMSCs and supported robust proliferation.

**Figure 4:**
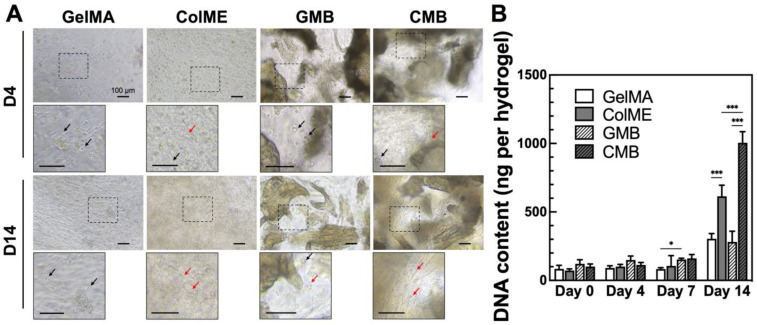
In vitro evaluation of hBMSCs morphology and proliferation in hydrogels: (**A**) Cell morphology and adhesion on day 4 and 14. Black arrow indicates nonadherent cells, while red arrow indicates adherent cells; (**B**) Quantitative analysis of DNA content over 14 days of culture (N = 6). All data are shown as mean ± standard deviation; *, *p* < 0.05 and ***, *p* < 0.001.

Next, to assess the effect of mDBMp on cell proliferation, hBMSCs were encapsulated in GelMA-based (GelMA, GMB) and ColME-based (ColME, CMB) hydrogels. By day 4, initial cell elongation and adhesion were observed in ColME and CMB hydrogels, whereas minimal elongation was noted in GelMA and GMB hydrogels (Figure 4A). Over 14 days, cell elongation was more pronounced in ColME and CMB hydrogels, indicative of improved cell–matrix interactions. DNA quantification revealed that, within 7 days, the overall increase in DNA content was less than 2.0-fold across all hydrogels, suggesting an early adaptation phase without significant cell death (Figure 4B). By day 14, the ColME hydrogel showed 2.0-fold higher DNA content than the GelMA hydrogel, while the CMB hydrogel was 3.6-fold greater than the GMB hydrogel. Notably, GelMA provides relatively fewer cell-binding motifs (e.g., RGD sequences) than ColME, which may explain the limited cell proliferation observed in GelMA-based hydrogels, even with mDBMp. Moreover, the DNA content in the CMB hydrogel was 1.6-fold higher than that in the ColME hydrogel, demonstrating the superior proliferation of hBMSCs in the ColME-based system with mDBMp compared to GelMA-based counterpart.

### 3.6. In Vitro Osteogenic Differentiation of hBMSCs in Hydrogels

We assessed the osteogenic differentiation of hBMSCs as detailed in Supplementary Materials S5. Calcium deposition was visualized by ARS staining, and alkaline phosphatase (ALP) activity was measured, indicating progression toward late-stage osteogenic differentiation within 14 days. These results confirm that hBMSCs can differentiate into osteogenic lineages.

**Figure 5:**
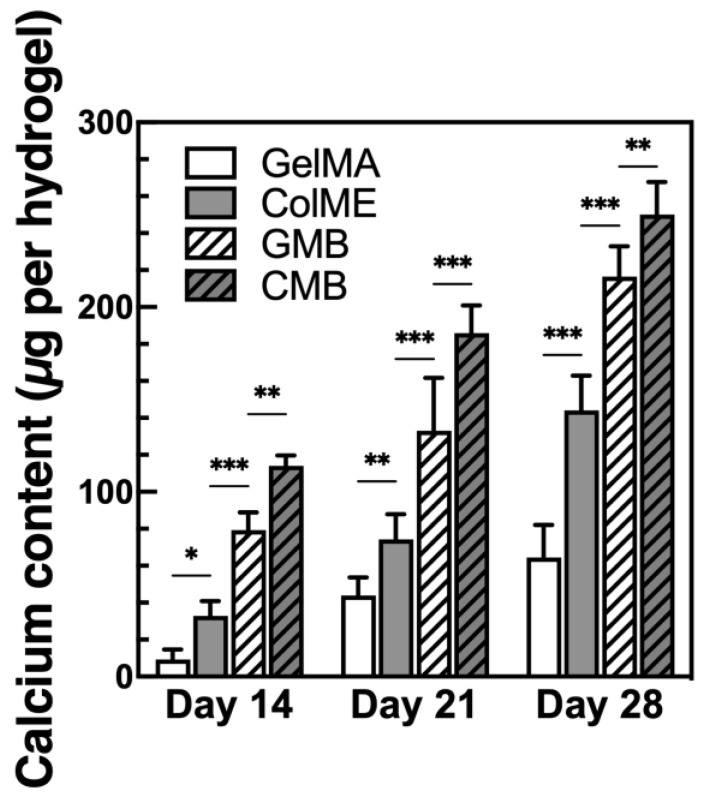
Mineralization of hBMSC lineage in hydrogels after 28 days of osteogenic induction (N = 6). All data are shown as mean ± standard deviation; *, *p* < 0.05, **, *p* < 0.01, and ***, *p* < 0.001.

To evaluate the ability of mDBMp-incorporated hydrogels to promote osteogenesis, hBMSCs were encapsulated in GelMA, ColME, GMB, and CMB hydrogels and cultured in osteogenic medium (OM) for 28 days. During induction, visible calcium deposition, indicative of osteoblast-mediated mineralization, was observed as dark regions under bright-field microscopy (see S6 in the Supplementary Materials). Notably, the GelMA hydrogel exhibited centralized nodules, while the ColME hydrogel displayed more uniform and extensive area of calcium deposition. In the composite hydrogels containing mDBMp (GMB and CMB hydrogels), visual assessment of calcium deposition was impeded by the presence of the particles, necessitating quantitative analysis of calcium content (Figure 5). By day 14, the calcium content in the ColME hydrogel (33 ± 8 µg) was 3.5-fold higher than that in the GelMA hydrogel (9 ± 5 µg). With the incorporation of mDBMp, the calcium content further increased to 79 ± 10 µg in the GMB hydrogel, an 8.5-fold increase relative to the GelMA hydrogel, and to 114 ± 6 µg in the CMB hydrogel, representing a 3.5-fold increase over the ColME hydrogel. By day 28, mineralization progressed further, calcium levels reached 65 ± 18 µg for the GelMA hydrogel, 144 ± 19 µg for the ColME hydrogel, 216 ± 17 µg for the GMB hydrogel, and 250 ± 18 µg for the CMB hydrogel. Overall, ColME-based hydrogels consistently showed greater mineralization than GelMA-based hydrogels, although mDBMp incorporation led both GMB and CMB hydrogels toward comparably high mineralization by day 28. While Figure 4B demonstrates higher cell proliferation in CMB than in GMB, the dominant factor driving mineralization appears to be the nucleation sites provided by mDBMp rather than the hydrogel matrix itself. These findings align with earlier studies showing that collagen hydrogels, especially with osteo-inductive growth factors, favor MSC differentiation and mineralization [54]. Our findings further indicate that mDBMps play a key role in enhancing mineralization, significantly improving the osteogenic performance of ColME-based hydrogels and compensating for the lower mineralization observed in GelMA-based hydrogels.

**Figure 6:**
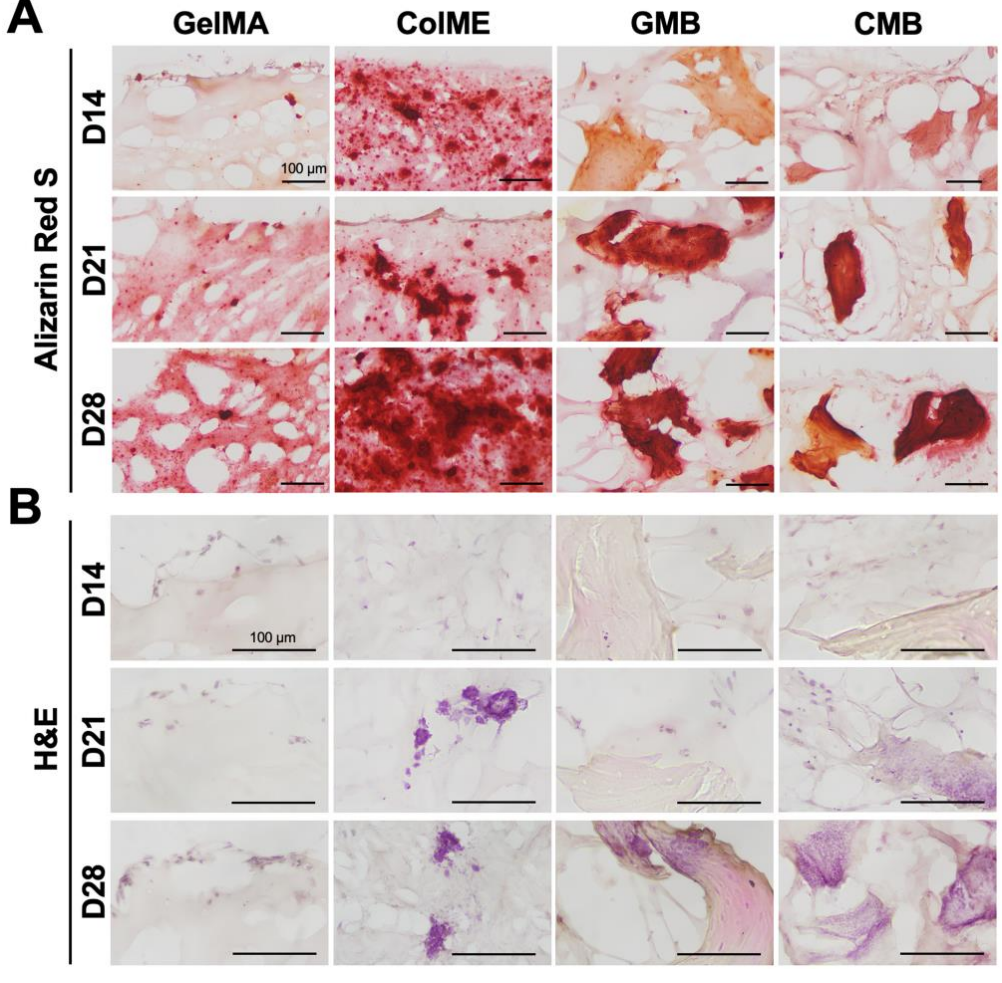
Histological evaluation of osteogenic induction in GelMA, ColME, GMB, and CMB hydrogels: (**A**) ARS-stained sections and (**B**) H&E-stained sections on day 14, 21, and 28.

Histological evaluation using ARS staining revealed calcium deposition throughout the cross-sections of all hydrogels (Figure 6A and Figure S7 in the Supplementary Materials). In the GelMA hydrogel, calcium nodules were confined to the surface, gradually spreading inward by day 28. Conversely, the ColME hydrogel already displayed nodules in both surface and deeper regions as early as day 14, with a marked increase in the deposition area by day 28. When mDBMps were incorporated, calcium deposition in the GMB hydrogel was concentrated around the particles. By day 28, closely interconnected nodules were apparent in deeper zones, indicating that mDBMp promotes mineralization in a three-dimensional microenvironment. In the CMB hydrogel, dense nodules were visible from day 14, suggesting an early onset of mineralization. Notably, the osteogenic potential of ColME facilitated the extension of calcium deposition from the nodule-rich mDBMp regions into the surrounding ColME matrix and, by day 28, calcium had become widely dispersed throughout the matrix.

H&E-stained sections revealed blue-purple staining of nucleic acids and pink staining of proteins and cytoplasm (Figure 6B and Figure S7 in the Supplementary Materials). In the GelMA hydrogel, cells were localized on the surface, aligning with the ARS staining pattern of calcium deposition. Conversely, the ColME hydrogel displayed adherent and scattered cells with visible nuclei and cytoplasm, indicating active cell–matrix interactions. Over time, noticeable cell clusters formed within the ColME hydrogel, especially on days 21 and 28. In the GMB hydrogel, no apparent cell attachment on mDBMp was observed before day 21. However, by day 28, cell clumps were clearly visible. The CMB hydrogel exhibited the most pronounced cell adhesion and clustering, with elongated cells observed in the ColME matrix as early as day 14 and abundant cells around mDBMp after day 21. This highlights the synergistic effect of ColME and mDBMp in promoting cell adhesion and nodule-induced differentiation. Overall, ColME-based hydrogels provided significant adhesive motifs for cells and facilitated the aggregation of cells from mDBMp, supporting three-dimensional differentiation.

From a biological standpoint, the maleic anhydride modification in ColME introduced anionic functional groups, particularly carboxylic acids, that lowered its isoelectric point (pI) to 3.5, as discussed in Section 3.1. Such anionic groups (PO_4_^3−^ and COOH) are known to attract calcium ions, which explains the superior osteogenesis potential and extent of mineralization in ColME compared to GelMA, as evidenced in the study [55]. Moreover, the development of ColME not only endowed it with anionic properties for improved calcium uptake and chelation, but also rendered it photocrosslinkable, a significant advancement over previous modification of non-photocrosslinkable collagen for enhanced mineralization [56]. Meanwhile, mDBMp contributed not only to cell aggregation and mineralization, but also to the mechanical reinforcement of the hydrogel matrix.

## 4. Conclusions

In this study, we developed a novel biomimetic hydrogel combining ColME and mDBMp, further reinforced with HAp. Our chemical modifications overcame the limitations of conventional collagen hydrogels by enhancing their mechanical performance through photocrosslinking. Modification of the particles enabled the effective integration of mDBMp into ColME, forming the CMB hydrogel with enhanced physiological stability and mechanical strength. Subsequent HAp mineralization confirmed that the CMB hydrogel efficiently supports the formation of pure HAp, suggesting strong potential for bone tissue engineering. In vitro, hBMSCs demonstrated superior proliferation within the CMB hydrogel compared to the GMB hydrogel. Under osteogenic induction, the CMB hydrogel exhibited the highest mineralization relative to GelMA, ColME, and GMB hydrogels. Overall, this bone-mimetic hydrogel, which comprises enriched ECM components, shows considerable promise for clinical bone regeneration. Based on these findings, future investigations using appropriate in vivo models of critical-sized bone defects are necessary to fully elucidate the new bone formation and osteointegration potential of the CMB hydrogel.

## Data Availability

Data are contained within the article or Supplementary Materials.

## Supplementary Materials

The following supporting information can be downloaded at: https://www.mdpi.com/article/10.3390/polym17070935/s1, Figure S1: Schematic illustration of the production of DBMp from native porcine bone; Figure S2: Schematic illustration of crosslinking in ColME–mDBMp hydrogels, forming the CMB composite hydrogel; Figure S3: Visual appearance of hydrogels after shrinkage in PBS at 37 °C; Figure S4: Cell viability and DNA content of hBMSCs encapsulated in 1% ColME hydrogel after 14 days of culture in complete medium; Figure S5: Osteogenic differentiation of hBMSCs over 14 days; Figure S6: Appearance of hBMSC lineage encapsulated in hydrogels under bright-field with osteogenic induction for 28 days; Figure S7: Broad-view histological analysis of osteogenic induction in GelMA, ColME, GMB, and CMB hydrogels. References [57,58,59] are cited in the Supplementary Materials.

## References

[1] Schemitsch E.H. (2017). Size matters: Defining critical in bone defect size!. J. Orthop. Trauma.

[2] Schmidt A.H. (2021). Autologous bone graft: Is it still the gold standard?. Injury.

[3] Chatelet M., Afota F., Savoldelli C. (2022). Review of bone graft and implant survival rate: A comparison between autogenous bone block versus guided bone regeneration. J. Stomatol. Oral Maxillofac. Surg..

[4] García-Gareta E., Coathup M.J., Blunn G.W. (2015). Osteoinduction of bone grafting materials for bone repair and regeneration. Bone.

[5] Fan L., Chen S., Yang M., Liu Y., Liu J. (2024). Metallic materials for bone repair. Adv. Healthc. Mater..

[6] Arambula-Maldonado R., Mequanint K. (2022). Carbon-based electrically conductive materials for bone repair and regeneration. Mater. Adv..

[7] Govindarajan D., Saravanan S., Sudhakar S., Vimalraj S. (2023). Graphene: A Multifaceted Carbon-Based Material for Bone Tissue Engineering Applications. ACS Omega.

[8] Rajendran A.K., Anthraper M.S.J., Hwang N.S., Rangasamy J. (2024). Osteogenesis and angiogenesis promoting bioactive ceramics. Mater. Sci. Eng. R Rep..

[9] Fama C., Kaye G.J., Flores R., Lopez C.D., Bekisz J.M., Torroni A., Tovar N., Coelho P.G., Witek L. (2021). Three-dimensionally-printed bioactive ceramic scaffolds: Construct effects on bone regeneration. J. Craniofacial Surg..

[10] Cheah C.W., Al-Namnam N.M., Lau M.N., Lim G.S., Raman R., Fairbairn P., Ngeow W.C. (2021). Synthetic material for bone, periodontal, and dental tissue regeneration: Where are we now, and where are we heading next?. Materials.

[11] Alvarez Echazu M.I., Perna O., Olivetti C.E., Antezana P.E., Municoy S., Tuttolomondo M.V., Galdopórpora J.M., Alvarez G.S., Olmedo D.G., Desimone M.F. (2022). Recent advances in synthetic and natural biomaterials-based therapy for bone defects. Macromol. Biosci..

[12] Lin X., Patil S., Gao Y.-G., Qian A. (2020). The bone extracellular matrix in bone formation and regeneration. Front. Pharmacol..

[13] Ferreira A.M., Gentile P., Chiono V., Ciardelli G. (2012). Collagen for bone tissue regeneration. Acta Biomater..

[14] Yue S., He H., Li B., Hou T. (2020). Hydrogel as a biomaterial for bone tissue engineering: A review. Nanomaterials.

[15] Samadian H., Maleki H., Allahyari Z., Jaymand M. (2020). Natural polymers-based light-induced hydrogels: Promising biomaterials for biomedical applications. Coord. Chem. Rev..

[16] Zhang Z., Liu W., Li D., Li G. (2007). Physicochemical properties of succinylated calfskin pepsin-solubilized collagen. Biosci. Biotechnol. Biochem..

[17] Drzewiecki K.E., Parmar A.S., Gaudet I.D., Branch J.R., Pike D.H., Nanda V., Shreiber D.I. (2014). Methacrylation induces rapid, temperature-dependent, reversible self-assembly of type-I collagen. Langmuir.

[18] Pamfil D., Nistor M.T., Zemljic L.F., Verestiuc L., Cazacu M., Vasile C. (2014). Preparation and characterization of methyl substituted maleic anhydride: Modified collagens destined for medical applications. Ind. Eng. Chem. Res..

[19] Shi H., Li Y., Xu K., Yin J. (2023). Advantages of photo-curable collagen-based cell-laden bioinks compared to methacrylated gelatin (GelMA) in digital light processing (DLP) and extrusion bioprinting. Mater. Today Bio.

[20] Hwangbo H., Lee H., Roh E.J., Kim W., Joshi H.P., Kwon S.Y., Choi U.Y., Han I.-B., Kim G.H. (2021). Bone tissue engineering via application of a collagen/hydroxyapatite 4D-printed biomimetic scaffold for spinal fusion. Appl. Phys. Rev..

[21] Wu Z., Bai J., Ge G., Wang T., Feng S., Ma Q., Liang X., Li W., Zhang W., Xu Y. (2022). Regulating macrophage polarization in high glucose microenvironment using lithium-modified bioglass-hydrogel for diabetic bone regeneration. Adv. Healthc. Mater..

[22] Wierzbicka A., Bartniak M., Waśko J., Kolesińska B., Grabarczyk J., Bociaga D. (2024). The Impact of Gelatin and Fish Collagen on Alginate Hydrogel Properties: A Comparative Study. Gels.

[23] Tabatabaei F., Moharamzadeh K., Tayebi L. (2020). Fibroblast encapsulation in gelatin methacryloyl (GelMA) versus collagen hydrogel as substrates for oral mucosa tissue engineering. J. Oral Biol. Craniofacial Res..

[24] Hoshikawa A., Nakayama Y., Matsuda T., Oda H., Nakamura K., Mabuchi K. (2006). Encapsulation of chondrocytes in photopolymerizable styrenated gelatin for cartilage tissue engineering. Tissue Eng..

[25] Chen C., Li Z., Xu C., Kang M., Lee C.S., Aghaloo T., Lee M. (2024). Self-Assembled Nanocomposite Hydrogels as Carriers for Demineralized Bone Matrix Particles and Enhanced Bone Repair. Adv. Healthc. Mater..

[26] Li D., Yang Z., Zhao X., Luo Y., Ou Y., Kang P., Tian M. (2021). A bone regeneration strategy via dual delivery of demineralized bone matrix powder and hypoxia-pretreated bone marrow stromal cells using an injectable self-healing hydrogel. J. Mater. Chem. B.

[27] Lee M.S., Lee D.H., Jeon J., Tae G., Shin Y.M., Yang H.S. (2020). Biofabrication and application of decellularized bone extracellular matrix for effective bone regeneration. J. Ind. Eng. Chem..

[28] Yu X., Jiang S., Li D., Shen S.G., Wang X., Lin K. (2024). Osteoimmunomodulatory bioinks for 3D bioprinting achieve complete regeneration of critical-sized bone defects. Compos. Part B Eng..

[29] Wu X., Huo Y., Ci Z., Wang Y., Xu W., Bai B., Hao J., Hu G., Yu M., Ren W. (2022). Biomimetic porous hydrogel scaffolds enabled vascular ingrowth and osteogenic differentiation for vascularized tissue-engineered bone regeneration. Appl. Mater. Today.

[30] Datta S., Rameshbabu A.P., Bankoti K., Roy M., Gupta C., Jana S., Das A.K., Sen R., Dhara S. (2021). Decellularized bone matrix/oleoyl chitosan derived supramolecular injectable hydrogel promotes efficient bone integration. Mater. Sci. Eng. C.

[31] Paduano F., Marrelli M., White L.J., Shakesheff K.M., Tatullo M. (2016). Odontogenic differentiation of human dental pulp stem cells on hydrogel scaffolds derived from decellularized bone extracellular matrix and collagen type I. PLoS ONE.

[32] Zhang X., He Y., Huang P., Jiang G., Zhang M., Yu F., Zhang W., Fu G., Wang Y., Li W. (2020). A novel mineralized high strength hydrogel for enhancing cell adhesion and promoting skull bone regeneration in situ. Compos. Part B Eng..

[33] Wu X., Zhang T., Hoff B., Suvarnapathaki S., Lantigua D., McCarthy C., Wu B., Camci-Unal G. (2021). Mineralized hydrogels induce bone regeneration in critical size cranial defects. Adv. Healthc. Mater..

[34] Wang L., Li D., Huang Y., Mao R., Zhang B., Luo F., Gu P., Song P., Ge X., Lu J. (2024). Bionic Mineralized 3D-Printed Scaffolds with Enhanced In Situ Mineralization for Cranial Bone Regeneration. Adv. Funct. Mater..

[35] Chen P.-H., Chen I.-H., Kao W.-H., Wu S.-Y., Tsai W.-B. (2024). Characterization and application of photocrosslinkable collagen maleate as bioink in extrusion-based 3D bioprinting. Biomater. Sci..

[36] Wu S.-Y., Tsai W.-B. (2023). Development of an In Situ Photo-Crosslinking Antimicrobial Collagen Hydrogel for the Treatment of Infected Wounds. Polymers.

[37] Lai T., Yu J., Tsai W. (2016). Gelatin methacrylate/carboxybetaine methacrylate hydrogels with tunable crosslinking for controlled drug release. J. Mater. Chem. B.

[38] Greco K., Francis L., Somasundaram M., Greco G., English N.R., Roether J.A., Boccaccini A.R., Sibbons P., Ansari T. (2015). Characterisation of porcine dermis scaffolds decellularised using a novel non-enzymatic method for biomedical applications. J. Biomater. Appl..

[39] AATB (2020). Standards for Tissue Banking.

[40] Bigham A., Dehghani S., Shafiei Z., Torabi Nezhad S. (2008). Xenogenic demineralized bone matrix and fresh autogenous cortical bone effects on experimental bone healing: Radiological, histopathological and biomechanical evaluation. J. Orthop. Traumatol..

[41] Sawkins M.J., Bowen W., Dhadda P., Markides H., Sidney L.E., Taylor A.J., Rose F.R., Badylak S.F., Shakesheff K.M., White L.J. (2013). Hydrogels derived from demineralized and decellularized bone extracellular matrix. Acta Biomater..

[42] Dozza B., Lesci I.G., Duchi S., Della Bella E., Martini L., Salamanna F., Falconi M., Cinotti S., Fini M., Lucarelli E. (2017). When size matters: Differences in demineralized bone matrix particles affect collagen structure, mesenchymal stem cell behavior, and osteogenic potential. J. Biomed. Mater. Res. Part A.

[43] Zhang X., Xu L., Wei S., Zhai M., Li J. (2013). Stimuli responsive deswelling of radiation synthesized collagen hydrogel in simulated physiological environment. J. Biomed. Mater. Res. Part A.

[44] Dong L., Liu Q., Gao Y., Jia H., Dai W., Guo L., Fan H., Fan Y., Zhang X. (2021). The effect of collagen hydrogels on chondrocyte behaviors through restricting the contraction of cell/hydrogel constructs. Regen. Biomater..

[45] Patrawalla N.Y., Kajave N.S., Kishore V. (2023). A comparative study of bone bioactivity and osteogenic potential of different bioceramics in methacrylated collagen hydrogels. J. Biomed. Mater. Res. Part A.

[46] Townsend J.M., Kiyotake E.A., Easley J.T., Seim III H.B., Stewart H.L., Fung K.-M., Detamore M.S. (2023). Comparison of a thiolated demineralized bone matrix hydrogel to a clinical product control for regeneration of large sheep cranial defects. Materialia.

[47] Conrad B., Yang F. (2022). Hydroxyapatite-coated gelatin microribbon scaffolds induce rapid endogenous cranial bone regeneration in vivo. Biomater. Adv..

[48] Nonoyama T. (2020). Robust hydrogel–bioceramics composite and its osteoconductive properties. Polym. J..

[49] Mansour S., El-Dek S., Ahmed M., Abd-Elwahab S., Ahmed M. (2016). Effect of preparation conditions on the nanostructure of hydroxyapatite and brushite phases. Appl. Nanosci..

[50] Šimková L., Gorodylova N.O., Dohnalová Ž., Šulcová P. (2018). Influence of precipitation conditions on the synthesis of hydroxyapatite. Ceram.-Silik..

[51] Moosavifar M., Parsaei H., Hosseini S., Mirmontazeri S.M., Ahadi R., Ahadian S., Engel F.B., Roshanbinfar K. (2022). Biomimetic Organic–Inorganic Nanocomposite Scaffolds to Regenerate Cranial Bone Defects in a Rat Animal Model. ACS Biomater. Sci. Eng..

[52] Gkioni K., Leeuwenburgh S.C., Douglas T.E., Mikos A.G., Jansen J.A. (2010). Mineralization of hydrogels for bone regeneration. Tissue Eng. Part B Rev..

[53] Pan Y., Zhao Y., Kuang R., Liu H., Sun D., Mao T., Jiang K., Yang X., Watanabe N., Mayo K.H. (2020). Injectable hydrogel-loaded nano-hydroxyapatite that improves bone regeneration and alveolar ridge promotion. Mater. Sci. Eng. C.

[54] Oh S.-A., Lee H.-Y., Lee J.H., Kim T.-H., Jang J.-H., Kim H.-W., Wall I. (2012). Collagen three-dimensional hydrogel matrix carrying basic fibroblast growth factor for the cultivation of mesenchymal stem cells and osteogenic differentiation. Tissue Eng. Part A.

[55] Kim S., Lee M. (2020). Rational design of hydrogels to enhance osteogenic potential. Chem. Mater..

[56] Jin W., Jin Y., Duan P., Wu H., Zhang L., Du Q., Pan H., Tang R., Shao C. (2022). Promotion of collagen mineralization and dentin repair by succinates. J. Mater. Chem. B.

[57] Reddy G.K., Enwemeka C.S. (1996). A simplified method for the analysis of hydroxyproline in biological tissues. Clin. Biochem..

[58] Neuman R.E., Logan M.A. (1950). The determination of hydroxyproline. J. Biol. Chem..

[59] Wang P.-Y., Tsai W.-B., Voelcker N.H. (2012). Screening of rat mesenchymal stem cell behaviour on polydimethylsiloxane stiffness gradients. Acta Biomater..

