# Supplementary material for: Development of a Photocrosslinkable Collagen–Bone Matrix Hydrogel for Bone Tissue Engineering
Source: Polymers (Basel). 2025 Mar 29;17(7):935. doi: 10.3390/polym17070935 (PMC11991309; doi:10.3390/polym17070935)
Supplement: Supplementary file 1 [file polymers-17-00935-s001.zip › polymers-3540638-supplementary.pdf]

Development of a Photocrosslinkable Collagen–Bone Matrix Hydrogel for Bone Tissue Engineering

Supplementary Materials

S1. Hydroxyproline Assay

To quantify the collagen concentration, hydroxyproline was measured to quantify the collagen and ColME concentration. The formation of chromophore from hydroxyproline was measured by adding Ehrlich's reagent [1]. In brief, 200 μL of collagen solution and 200 μL of 2N NaOH were combined in a glass container capable of enduring hydrolytic reaction (120°C, 17 psi for 90 minutes), followed by neutralization with 200 μL of 2N HCl. After cooling the samples, diluted the samples with 2.5 mM citric acid (Sigma-Aldrich, USA) with 5 mM Na_2HPO_4 (Sigma-Aldrich, USA) at pH 6.0. The standard curve was prepared by trans-4-hydroxy-L-proline (Sigma-Aldrich, USA). The standard chemicals and samples were transferred into 96-well plate for 50 μL/well and added with 75 μL of chloramine-T reagent (1.0% w/v of chloramine-T in deionized water then diluted with equal volume of citrate phosphate buffer) (Sigma-Aldrich, USA) for 15 minutes at room temperature to form pyrrole. During this period, Ehrlich reagent was freshly prepared from 560 mg of 4-(Dimethylamino)benzaldehyde (DMAB; Sigma-Aldrich, USA) in 1200 μL 70% $HClO_4$ (Sigma-Aldrich, USA) and 6.8 mL of deionized water. Subsequently, 75 μL of the Ehrlich reagent was added to each well and incubated at 70°C for 20 minutes. Measurement of chromophore was conducted by a microplate reader (BioTek Synergy H1, USA) at the absorbance of 560 nm. Finally, collagen concentration was calculated by the average composition of hydroxyproline in collagen type I, approximately 13-14% by mass in bovine tendon, so we assumed approximately 13.5% [2].

S2. Preparation of Demineralized and Decellularized Bone Matrix Particles (DBMp)

The illustration of preparation of demineralized and decellularized bone matrix particles (DBMp) from native porcine bone. The process involved removal of muscles and tendons using a scalpel and hydrogen peroxide (H_2O_2; Sigma-Aldrich, USA), primary fragmentation of the bone, demineralization, multiple cycles of grinding and sieving, and subsequent decellularization.

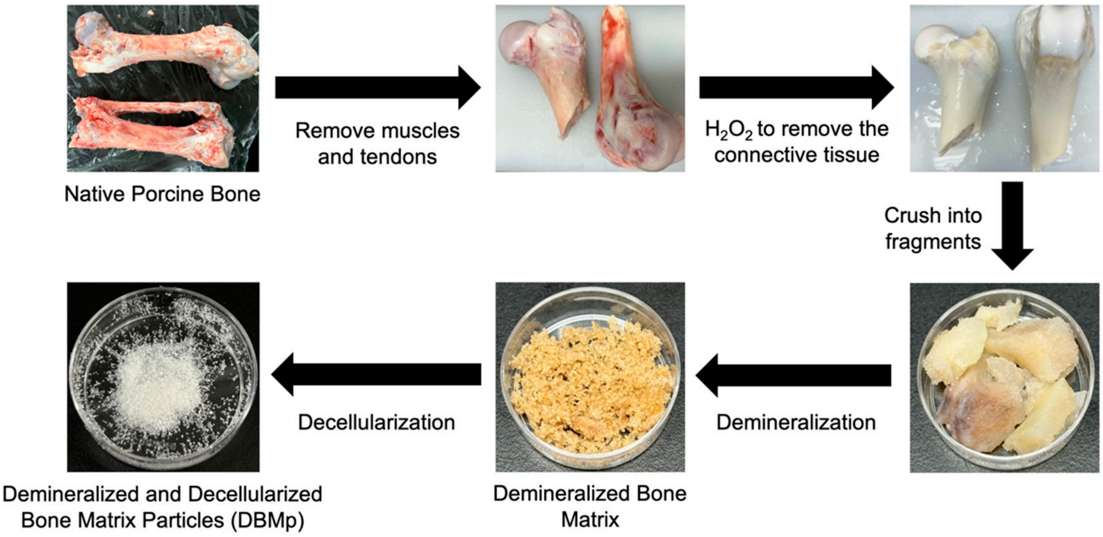

Figure S1. Schematic illustration of the production of DBMp from native porcine bone.

S3. Physiological Stability of ColME-Based Hydrogels

To evaluate hydrogel properties under hydration, 1% ColME was mixed with either 5% demineralized and decellularized bone matrix particles (DBMp) or 5% maleic anhydride–modified demineralized and decellularized porcine bone matrix particles (mDBMp), along with 0.06% lithium phenyl-2,4,6-trimethylbenzoylphosphinate (LAP; Sigma-Aldrich, USA). The hydrogel precursor was photocrosslinked using UV irradiation (10 mW/cm^2 at 365 nm) for 1.5 minutes, yielding CMB hydrogels, as shown in Figure S2. The CMB hydrogels were then immersed in phosphate-buffered saline (PBS) along with ColME and CB hydrogels at 37°C, as shown in Figure S3. Volume change was determined by the average height and diameter using NIH ImageJ.

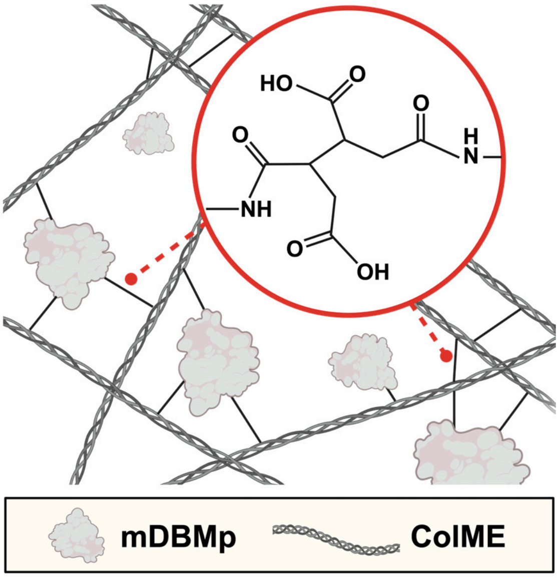

Figure S2. Schematic illustration of crosslinking in ColME–mDBMp hydrogels, forming the CMB composite hydrogel.

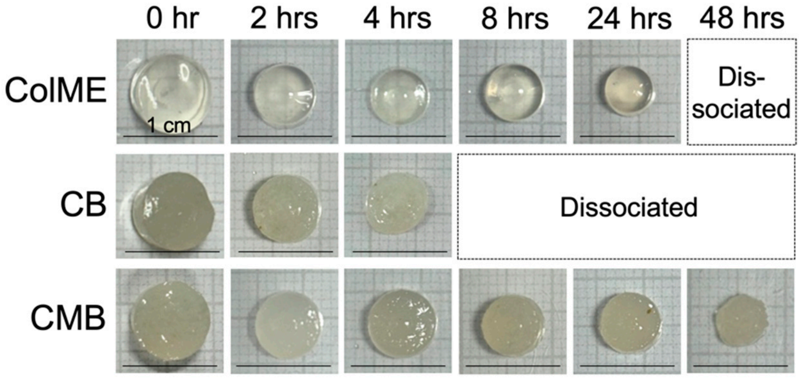

Figure S3. Visual appearance of hydrogels after shrinkage in PBS at 37°C. ColME=1% ColME hydrogel. CB=1% ColME+5% DBMp hydrogel. CMB=1% ColME+5% mDBMp hydrogel.

S4. Cell Viability and Proliferation of hBMSCs in ColME Hydrogel

We evaluated the cytocompatibility and proliferation of immortalized human bone marrow mesenchymal stem cells (hBMSCs, passages 15–25, T0523 from Applied biological materials, Canada) encapsulated in a basal 1% ColME hydrogel. Briefly, hBMSCs (1×10^6 cells/mL) were mixed with a 1% ColME hydrogel precursor containing 0.06% LAP, with a total volume of 150 µL. The hydrogel was photocrosslinked by UV irradiation (10 mW/cm^2) for 1 minute and then cultured for 14 days in complete medium (α-MEM from Gibco, USA; 10% FBS from Peak Serum, USA; and 1% antibiotic-antimycotic solution from Capricorn, Germany).

Hydrogel samples were washed twice with PBS 4 hours after photocrosslinking on day 0 and collected on days 4, 7, and 14. Cell viability was assessed using LIVE/DEAD cell imaging on an inverted fluorescence microscope (IX71, Olympus, Japan) with exposure times not exceeding 50 ms, following the manufacturer’s protocol. For proliferation analysis, DNA content was quantified as follows: samples were digested with a solution containing 6.25 mg papain (Sigma-Aldrich, USA) and 78.8 mg L-cysteine dihydrochloride (Sigma-Aldrich, USA) dissolved in 50 mL phosphate-buffered EDTA (pH 6.5) at 60°C for 24 hours, and then analyzed using the PicoGreen dsDNA assay according to the manufacturer’s instructions.

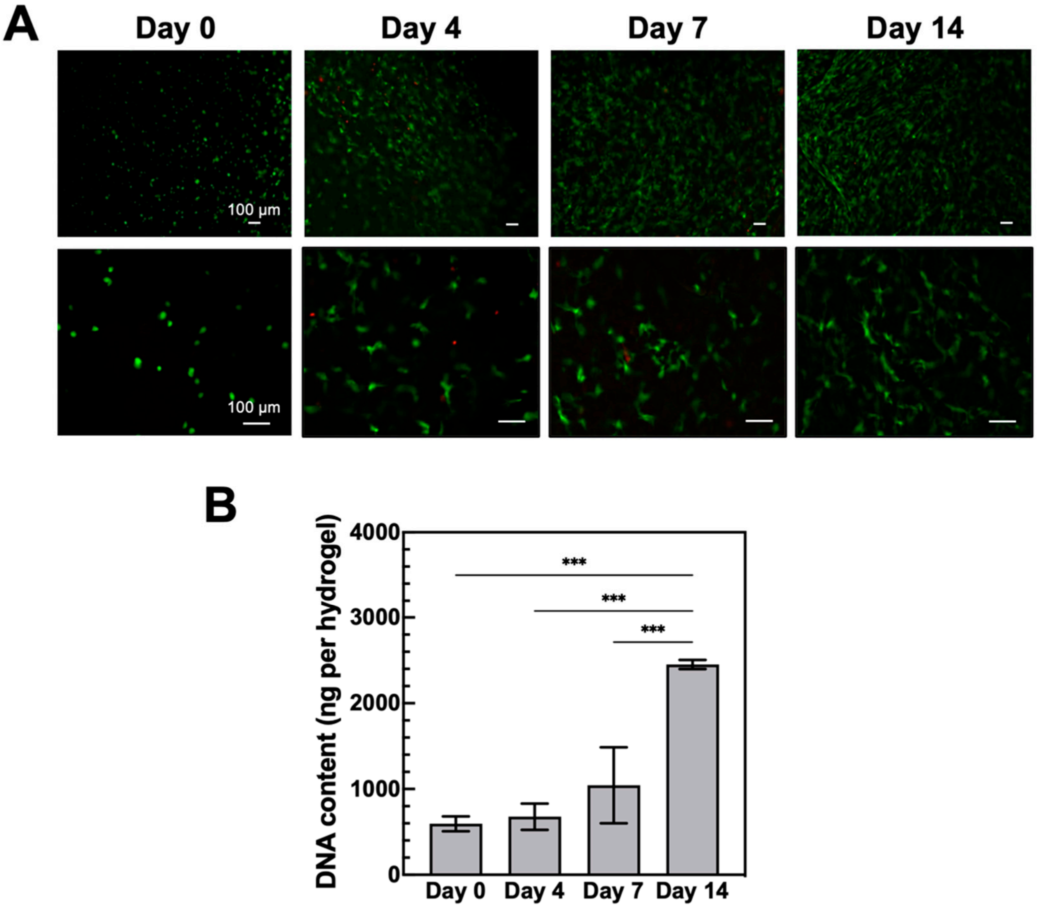

Figure S4. (**A**) LIVE/DEAD images and (**B**) DNA content of hBMSCs encapsulated in ColME after 14 days of culture in complete medium (N=3). Green=Live cells. Red=Dead cells.

S5. Osteogenic Differentiation of hBMSCs

hBMSCs was performed osteogenic differentiation on TCPS over 14 days. Briefly, hBMSCs were seeded at a density of 20,000 cells/cm^2 in 6-well and 96-well plates and cultured in a complete medium of α-MEM supplemented with 10% FBS for 2 days based on the previous research [3]. Subsequently, the medium was replaced with osteogenic medium (OM; α-MEM supplemented with 30 nM dexamethasone, 10 mM β-glycerophosphate, 50 μM L-ascorbic acid 2-phosphate, and 10% FBS) for a duration of 14 days, with half of the medium refreshed every 3 days.

The alkaline phosphatase (ALP) activity of hBMSCs was quantified after 7 and 14 days of osteogenic differentiation. Specifically, the medium was removed, and the cells were washed twice with PBS. The cells were then lysed with 0.2% Triton X-100 under gentle shaking for 20 minutes to obtain the cell lysate. A standard curve was prepared using p-nitrophenol (p-NP; Sigma-Aldrich, USA) dissolved in deionized water, with concentrations ranging from 0 to 100 μM. For the assay, 50 μL of the cell lysate or standard solution was loaded into a 96-well plate, followed by the addition of 100 μL of the working solution containing 4-nitrophenyl phosphate disodium salt hexahydrate (p-NPP; Sigma-Aldrich, USA). The reaction was carried out at 37°C for one hour, during which p-NPP was hydrolyzed by ALP into a yellow-colored product (p-NP). The absorbance of the reaction product was measured at

405 nm using a microplate reader to quantify ALP activity. To assess calcium deposition and mineralization in the well plate, the cells were first washed twice with PBS to remove any residual medium. They were then fixed with 4% paraformaldehyde (Sigma-Aldrich, USA) for 5 minutes at room temperature. Following fixation, the cells were stained with Alizarin Red S (ARS; Sigma-Aldrich, USA) solution for 20 minutes to visualize calcium deposits. After staining, the wells were washed three times with deionized water under gentle shaking for 5 minutes each to remove excess stain.

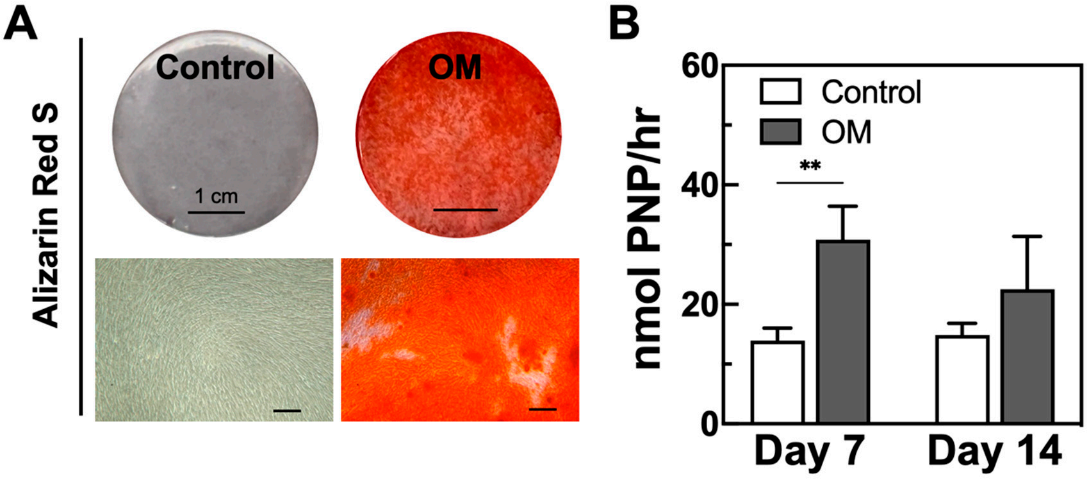

Figure S5. Osteogenic differentiation of hBMSCs over 14 days: (**A**) Macroscopic and microscopic images of cells with ARS stained; (**B**) ALP activity quantified from cells cultured in 96-well plate (N=4). Control refers to hBMSCs cultured in complete medium, while OM refers to hBMSCs cultured in osteogenic medium.

S6. Mineralization of hBMSCs Encapsulated in Hydrogels

hBMSCs were encapsulated in 50 µL of hydrogel precursor at a density of 2×10^6 cells/mL and photocrosslinked using UV irradiation (365 nm at 10 mW/cm^2) for 1.5 minutes to achieve complete gelation for all hydrogels. After 3 days in complete medium, the culture medium was replaced with osteogenic medium (OM). Mineralization was then evaluated via optical microscopy (Nikon Eclipse TS2, Japan). In 5% GelMA hydrogel, a centralized mineralized nodule appeared on day 14 and gradually dispersed by day 28. In contrast, 1% ColME hydrogel exhibited uniform calcium deposition throughout, resulting in increased density on day 28. These observations suggest that hBMSCs follow distinct differentiation patterns in GelMA versus ColME hydrogels. In hydrogels incorporating mDBMp (GMB and CMB hydrogels), calcium-rich areas were indistinguishable due to particle interference.

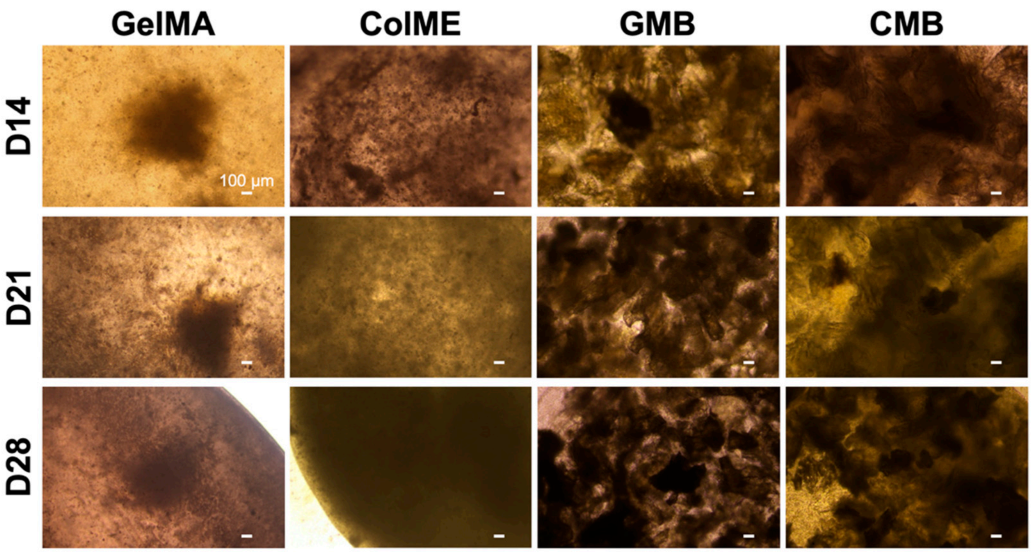

Figure S6. Appearance of hBMSCs lineage encapsulated in hydrogels under bright-field with osteogenic induction for 28 days.

S7. Histological Images of ARS– and H&E–Stained Sections

Hydrogels are considered soft tissue-like samples, making them suitable for dehydration and cryosectioning. To prevent structural collapse from ice crystals and minimize rupture during sectioning, the hydrogels were dehydrated in a gradient of sucrose (Sigma-Aldrich, USA) solutions: 10%, 20%, and 30% for 2, 3, and 14 hours, respectively. An OCT compound (Fisher HealthCare, USA) base was prepared firstly by freezing it with liquid nitrogen. Subsequently, additional OCT compound was gently poured over the frozen base, and the hydrogel was carefully placed into the compound. Care was taken to avoid generating bubbles during the entire process. Finally, the sample was rapidly frozen in a liquid nitrogen container again. Cryosectioning was performed using a cryostat microtome (CM1900; Leica, Germany), producing sections with a thickness of 20 μm.

Two histochemical staining, Alizarin Red S (ARS) and hematoxylin & eosin (H&E; Sigma-Aldrich, USA), were applied to each section, with the ARS and eosin solutions freshly prepared. To ensure adherence of the cryosectioned samples to the slides, an additional drying step was carried out at 55°C for 30 minutes. For ARS staining, the OCT compound was removed by washing the slides with deionized water for 1 minute. The slides were then stained with ARS solution for 45 seconds to assess calcium deposition and mineralization, followed by a quick rinse in deionized water (1 dip). Dehydration was performed sequentially using a xylene-acetone mixture (1:1 by volume) for 1 minute, followed by pure xylene for another minute. The slides were finally mounted with DPX mountant (Sigma-Aldrich, USA). For H&E staining, OCT compound was washed out with deionized water for 1 minute and then stained with hematoxylin to visualize nuclei for 30 seconds, followed by a quick rinse in deionized water (1 dip). The extracellular matrix was then stained with eosin for 1 minute, followed by another rinse in deionized water (1 dip). Dehydration was carried out sequentially in a xylene-acetone mixture (1:1 by volume) for 1 minute, followed by pure xylene for additional 1 minute. Finally, the slides were mounted with DPX mountant.

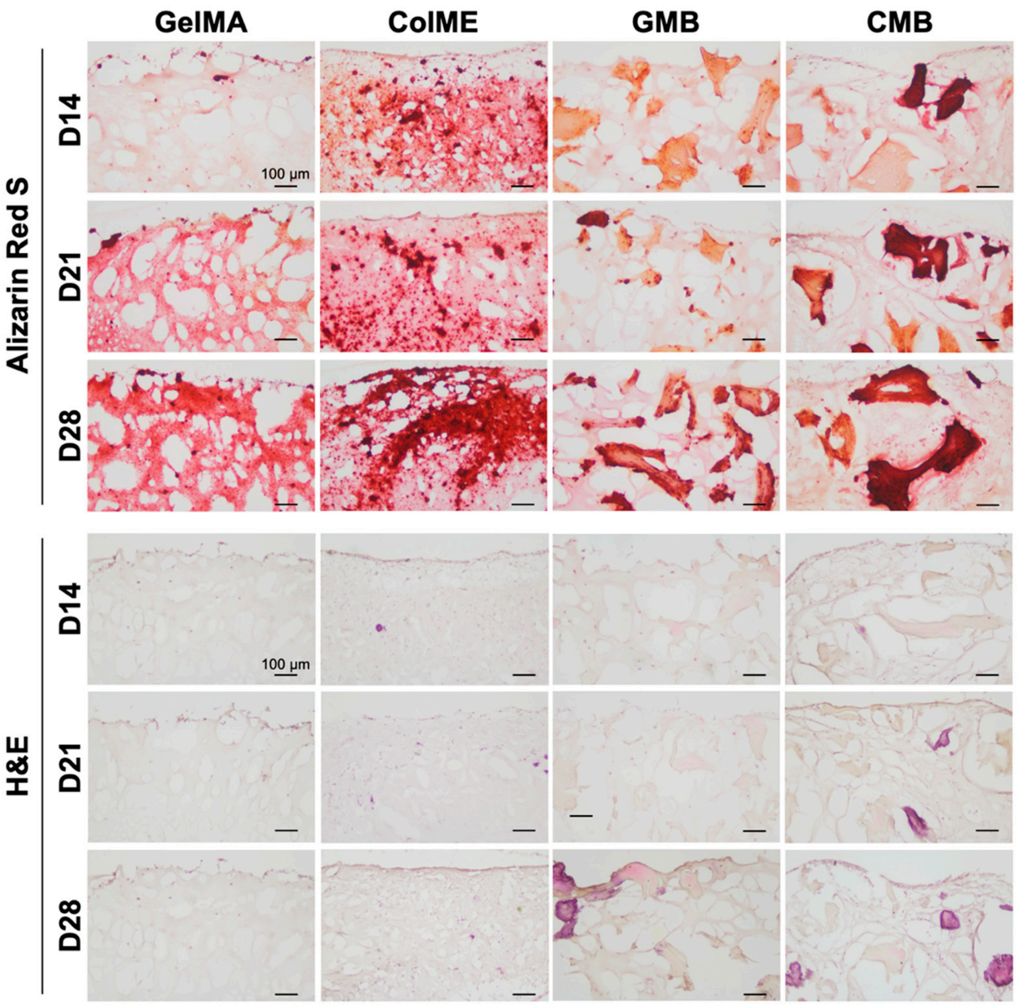

Figure S7. Broad-view histological analysis of osteogenic induction in GelMA, ColME, GMB, and CMB hydrogels: (**A**) ARS-stained sections and (**B**) H&E-stained sections on day 14, 21, and 28.

References

1. Reddy, G.K.; Enwemeka, C.S. A simplified method for the analysis of hydroxyproline in biological tissues. *Clinical biochemistry* **1996**, *29*, 225-229.
2. Neuman, R.E.; Logan, M.A. The determination of hydroxyproline. *J Biol Chem* **1950**, *184*, 299-306.
3. Wang, P.-Y.; Tsai, W.-B.; Voelcker, N.H. Screening of rat mesenchymal stem cell behaviour on polydimethylsiloxane stiffness gradients. *Acta biomaterialia* **2012**, *8*, 519-530.
